# The Relationship between Variola and Vaccinia

**Published:** 1898-05-14

**Authors:** 


### The Relationship Between Variola and Vaccinia.

It may appear to some to be very like flogging a dead horse to discuss again the question of the relationship between variola and vaccinia, for it may be said—and with considerable justice—that the thing is settled, and that vaccinia is but a modification of small-pox. Nevertheless, settled as it may be, we think that Dr. Copeman has done well to deal with it fully over again in the Milroy lectures, for there can be but little doubt that there are scores of well-informed people, including no small number of medical men, who, if asked offhand, What is vaccinia? would answer: A disease of the cow which, when inoculated into man, protects him against small-pox.

We do not want to quarrel with the definition, which, indeed, has not only been current among the multitude for the greater part of the century, but has to some extent been driven home by legislation which has emphasised the distinction between the two diseases by making inoculation with the small-pox illegal, while on the other hand it made vaccination with vaccinia compulsory. The fact also is obvious enough that there is the greatest possible difference between these two manifestations of disease, for small-pox is a terribly fatal malady, while vaccinia is but a trifling affair. The real question, however, is this: Is vaccinia a disease of itself, independent and specific; a disease of cows, but capable of inoculation into man, or is it merely a bovine manifestation of human small-pox? The importance of the answer to this question is manifest. It was all very well for people at the beginning of the century to be told that vaccination was protective against small-pox; people in those days believed or did not believe, and there was an end of it. But nowadays we want to know the reason why, and on the hypothesis that vaccinia is a specific disease of the cow no explanation is forthcoming. We must take the bald fact as it stands, and in accepting it we must recognise that the fact rests upon nothing but empirical observation, and leads to nothing but empirical prophylaxis; a prophylaxis powerful enough, but barren as regards all teaching for the future.

If, however, vaccinia is but the bovine manifestation of human small-pox, its protective influence against the real malady falls into line with all that modern science has taught us in regard to the protective effects of modified and attenuated cultures of known pathogenic organisms, and we may fairly urge the performance of vaccination, not as a "Jennerian rite," as it is sarcastically called by the anti-vaccinists, but as a scientific endeavour to obtain protection in a reasonable way against a most fatal and disfiguring disease. Dr. Monckton Copeman marshals his facts in a logical manner, and his conclusion is perfectly definite. What he says is this: "If, as I believe, it can be conclusively proved that small-pox lymph, by passing through the system of the calf, can be so altered in character as to become deprived of its power of causing a generalised eruption, while inducing at the site of inoculation a vesicle indistinguishable from a typical vaccine vesicle, and, more important still, if it be shown that when transferred again to man it has by such treatment completely lost its former power to produce a general disease, it may fairly be asserted that cow-pox—or rather that artificially inoculated form of the disease which we call vaccinia—is nothing more nor less than variola modified by transmission through the bovine animal."

There are those, no doubt, who will be willing to say, What does it matter so that we get the protection from small-pox which vaccination will give? This, however, is to take but a limited and unimaginative view of medical science. Each fact gained should be the stepping-stone to something else, something better and more useful still, and the more definitely we can regard vaccinia as a modification of small-pox—in other words, the more sure we can feel that virulent small-pox can, by comparatively simple means, be so modified as to become at the same time protective and innocuous—the wider seem to be the possibilities of progress in the future. If the protective influence of vaccination is a mere accident, having no relation to the natural history of the virus of the disease, we are without a clue to future developments; but if the virus is a microbe, as the researches of Dr. Copeman seem to show to be the case, we ought not to be far from the production of an appropriate anti-toxin by which the disease may be deprived of many of its terrors; while if we are to accept as a broad generalisation that microbic infections can be modified by passing them through other animals, as human small-pox is modified by passing it through the calf, a very wide vista of prophylactic measures opens out before the eye of the scientific imagination.

